# Association of body roundness index with abdominal aortic calcification among middle aged and elderly population: findings from NHANES

**DOI:** 10.3389/fcvm.2024.1475579

**Published:** 2024-10-10

**Authors:** Ji Wu, Daojun Lu, Xiang Chen

**Affiliations:** ^1^ Department of Thyroid Breast Surgery, The Affiliated Yixing Hospital of Jiangsu University, Wuxi, China; ^2^ Department of Hand and Foot Surgery, Affiliated Huishan Hospital of Xinglin College, Nantong University, Wuxi Huishan District People's Hospital, Wuxi, China

**Keywords:** BRI, AAC, elderly, middle age, NHANES, abdominal obesity

## Abstract

**Aim:**

We aim to investigate the association between body roundness index (BRI) and abdominal aortic calcification (AAC) among middle aged and elderly US residents.

**Methods:**

This cross-sectional study used data from the National Health and Nutrition Examination Survey (NHANES) 2013–2014 cycle, including 3,079 middle-aged and elderly participants aged 40 and above. AAC scores for these participants were assessed using dual-energy x-ray absorptiometry (DXA). BRI was calculated from participants’ height and waist circumference, with all measurements conducted by trained surveyors using standardized methods. The relationship between BRI and AAC was analyzed using weighted multivariate logistic regression, adjusting for confounding variable. Additionally, restricted cubic splines (RCS) analysis was also employed.

**Results:**

We found that those with AAC were significantly older and had a higher prevalence of smoking and chronic kidney disease (CKD) prevalence compared to those without AAC. Using weighted multivariable logistic regression, we determined that an increase of one unit in BRI was associated with a 22% higher risk of AAC. Additionally, higher BRI quartiles (Q2, Q3, Q4) showed significantly increased risks of AAC compared to the lowest quartile. Visualization using RCS indicated a gradual increase in AAC risk with higher BRI, which plateaued beyond a BRI of 7.2. This relationship was significant across different age and gender group.

**Conclusion:**

There is a positive association between abdominal obesity (as measured by BRI) and AAC in the middle-aged and elderly population. This suggests the impact of abdominal obesity on vascular health and that this factor should be considered in public health strategies.

## Introduction

Abdominal aortic calcification (AAC) is a common degenerative condition in the elderly (1, 2). About 20% of people over 60 years-old with AAC (3–5). The pathological characteristics of AAC include atherosclerosis and reduced elasticity of the abdominal aorta. To some extent, arterial calcification is a marker of aging, and AAC can reflect the overall state of arterial calcification throughout the body (6). This is significant for predicting adverse cardiovascular and cerebrovascular events. Besides elderly, there are many risk factors that can lead to arterial calcification (7). For example, in hypertensive patients whose blood pressure is not controlled within the normal range over a long period, the arterial walls are affected by the high blood pressure, greatly increasing the risk of AAC (8, 9). Individuals with obesity often have diabetes and hyperlipidemia, hyperlipidemia can cause vascular stiffening, which also increases the risk of AAC. In diabetes, abnormal fat metabolism leads to increased fat deposition on the vascular walls, thereby inducing AAC.

Obesity has become a major public health issue worldwide (10). Studies have shown that deaths related to overweight and obesity account for 11.1% of deaths from non-communicable diseases (11, 12). Increasing research confirms that abdominal obesity poses a greater risk (13, 14). Abdominal obesity, characterized by excessive fat accumulation in the abdomen leading to increased waist circumference, significantly raises the risk of cardiovascular and cerebrovascular diseases (15). Causes of abdominal obesity include prolonged sedentary behavior and genetic factors, and it needs to be controlled through diet and physical exercise (16). Waist circumference and hip circumference are often used to compensate for the inability of BMI to reflect body fat distribution. However, these measures cannot account for the larger waist or hip circumference in taller individuals. Therefore, an index that comprehensively assesses both body height and width is needed. In 2013, Thomas developed the Body roundness index (BRI) as a method to predict body fat percentage and visceral adipose tissue percentage, thereby predicting overall health risk (17).

However, relationship of abdominal obesity with AAC is still not very clear. We aim to investigate the relationship between abdominal obesity, reflected by the BRI, and AAC in the middle-aged and elderly population.

## Methods

### Study population

**Figure 1:**
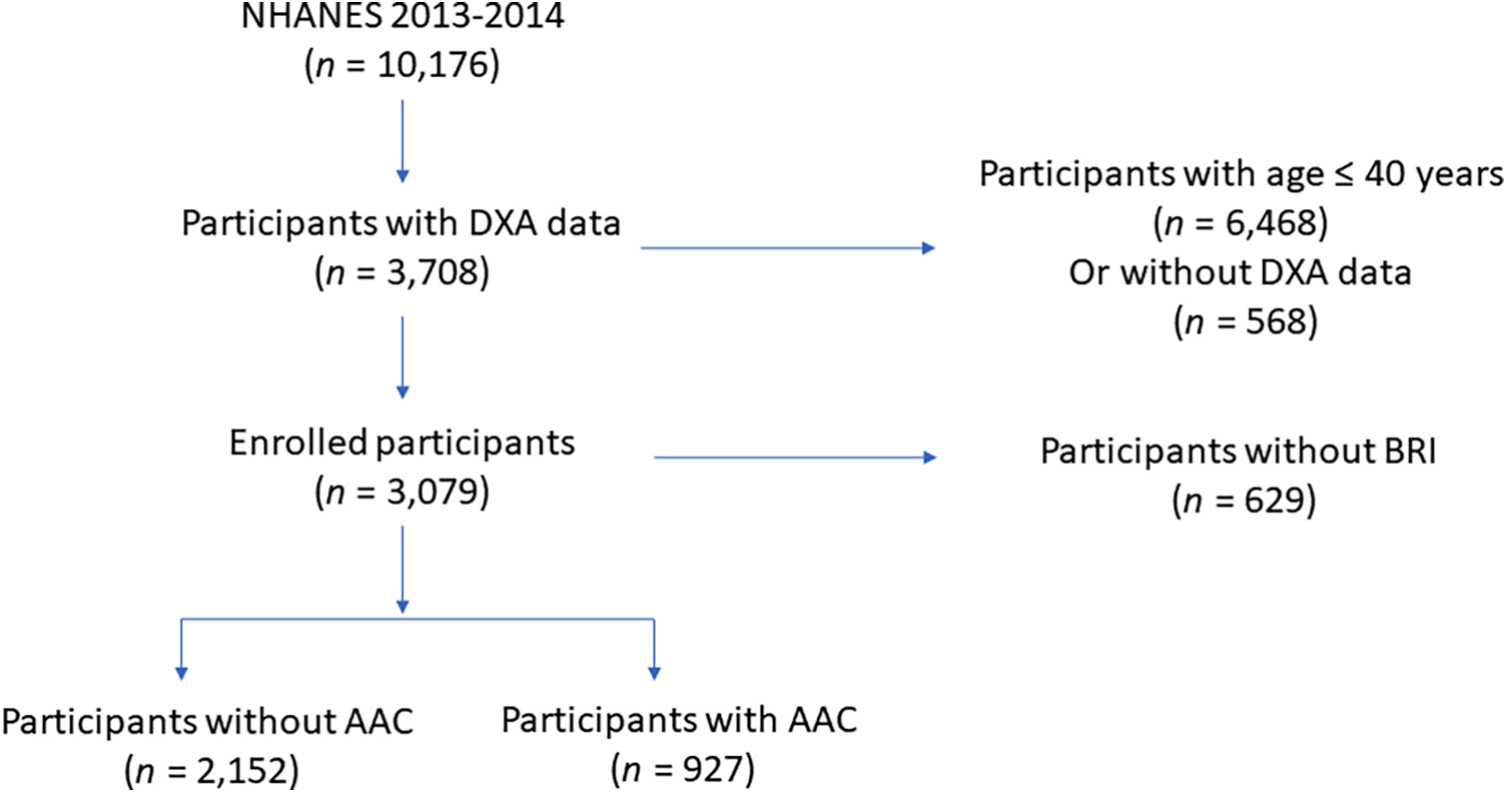
Study population enrollment.

All of the enrolled participants were from National Health and Nutrition Examination Survey (NHANES), which aims to investigate the overall health and nutrition status of residents in US. Due to AAC scores can only be obtained in study circle of NHANES 2013–2014 and participants under the age of 40 lacked AAC scores data, we initially included 3,708 participants aged over 40 years old in NHANES 2013–2014 with AAC score eligible. Moreover, there were 69 participants without BRI measurement were excluded. After removing 69 participants with missing BRI measurement, 3,079 eligible middle aged and elderly participants were ultimately included in the present study to study the association between BRI and AAC. Population enrollment can be found in Figure 1.

### BRI measurement

In NHANES, participants’ body height and waist circumference are measured by trained surveyors using standardized measurement methods. All measurement data undergo quality control and standardization processes to ensure accuracy and comparability of results. Consistent with previous studies, the formula for calculating BRI is as follows (17):

$$\text{BRI}=364.2-365.5\times \;\sqrt{1-{\left(\frac{\frac{\text{WC}}{2\pi }}{\;0.5\;\times \text{BH}}\right)}^{2}}$$

### AAC scores

Dual-energy x-ray absorptiometry (DXA) was adopted to evaluate AAC. The measurements were derived from a lateral scan focused on the lumbar spine (vertebrae L1–L4) and were assessed using the Kauppila score system, spaning from 0 to 24, classifying AAC into absence of calcification (AAC = 0), moderate calcification (AAC > 0–6), and severe calcification (AAC > 6) categories. A Kauppila score surpassing 6 indicates substantial calcification, aligning with earlier research findings. Exclusions from DXA scanning in the NHANES survey encompassed individuals under 40 years old, pregnant women, those weighing over 450 pounds, and participants who had consumed barium in the previous week. DXA machines are widely available in clinical settings, this widespread availability makes it relatively easy to incorporate arterial calcification measurements into existing practices. DXA is generally more cost-effective compared to advanced imaging techniques like CT or MRI. Moreover, DXA employs low-dose x-rays, resulting in minimal radiation exposure compared to other imaging techniques like CT scans. However, DXA may have lower sensitivity compared to other imaging modalities like CT in detecting early or subtle arterial calcification. Besides, in areas where bone and arteries are closely aligned, such as the spine or pelvis, the overlap of bone and arterial calcification can complicate the interpretation of DXA results, leading to potential inaccuracies.

### Covariates

Covariates were included in statistical analyses to control for potential confounding factors. We directly obtained participants’ demographical information through standardized demographic questionnaire. Blood pressure measurements was conducted by experienced examiners. Hypertensive status was categorized based on self-reported diagnosis, average systolic and diastolic blood pressure (140/90 mmHg), medication use, or laboratory tests. BMI was calculated using measured height and weight (kg/m^2^). Diabetes status was categorized as diabetic or non-diabetic based on self-reported diagnosis, medication use, or laboratory tests. Chronic kidney disease is categorized into five stages according to present clinical guidelines. Blood samples were obtained from participants after they had fasted for at least 8 h to determine the blood glucose, lipid, and other hematological parameters. Diabetes was assessed based on both historical diagnoses and newly identified cases. Participants with a fasting plasma glucose level of 7.0 mmol/L or higher were classified as having newly diagnosed diabetes. Moreover, Participants with an HbA1c level of 6.5% or higher were also diagnosed as participants with diabetes. We believe that retaining the original format of the data may more accurately reflect the actual situation. Similar to the weighted analysis methods used in previous research, we did not impute or exclude missing data.

### Statistical methods

Data were presented as weighted means with corresponding 95% CI, or weighted proportions with 95% CI. Each participant was given a specific sampling weight, primary sampling unit (PSU), and stratum to ensure the production of accurate national estimates. The WTMEC2YR dataset contains the survey weights for all participants who underwent body measurements at a Mobile Exam Center. For our analysis, we calculated the sample weight applied these survey weights in our statistical analyses to generate estimates that accurately reflect the U.S. population. The population included in the present study represents 2,213,412 individuals in US. Baseline characteristics among continuous variables were compared using the adjusted Wald test, and for categorical variables, the Rao-Scott *χ*^2^ test was employed. The association between BRI and AAC was examined using weighted multivariate logistic analysis. Covariates including age, sex, and race/ethnicity, income, education levels, BMI, smoking, drinking, hypertension, CKD, eGFR, HbA1c, serum phosphorus, serum calcium, serum albumin were adjusted in logistic and restricted cubic splines (RCS) analysis. Moreover, RCS analysis was also employed. Statistical analyses were conducted using R, with significance set at two tailed *p* < 0.05.

## Results

### Baseline demographical and clinical characteristics

A total of 3,079 middle-aged and elderly participants over 40 years. Overall, participants over 60 years accounted for approximately 40.28% of the sample, with males constituting about 48.25%. According to the baseline data, participants with AAC were significantly older than those without AAC. However, there were no significant differences in gender composition among two groups. Similarly, no notable differences were observed regarding education level and alcohol consumption. Notably, smokers was more in the AAC group. Additionally, it is well known that AAC is significantly associated with chronic kidney disease (CKD); the prevalence of CKD was significantly higher in the AAC group. Furthermore, we collected detailed demographic data and comprehensive hematological indicators for both groups, as shown in Table 1.

**Table 1:** Demographical characteristics of the study population grouped by AAC.

Variables	Overall (*n* = 3,079)	Non-AAC (*n* = 2,152)	AAC (*n* = 927)	*P* value
Age, years				<0.001***
40–49 years	30.23 [25.35, 35.10]	36.30 [34.37, 38.23]	15.14 [9.91, 20.37]	
50–59 years	29.50 [25.32, 33.67]	31.71 [28.42, 35.01]	23.98 [19.24, 28.73]	
60–69 years	22.57 [18.66, 26.48]	21.76 [19.35, 24.16]	24.59 [20.34, 28.84]	
>69 years	17.71 [15.67, 19.74]	10.23 [8.47, 12.00]	36.28 [30.74, 41.83]	
Sex-male,%	48.25 [41.70, 54.79]	48.01 [45.76, 50.25]	48.84 [46.28, 51.41]	0.671
Education level,%				0.620
High school or above	94.88 [82.66, 107.11]	95.08 [93.50, 96.65]	94.45 [91.92, 96.98]	
Below high school	5.10 [3.70, 6.51]	4.92 [3.35, 6.50]	5.55 [3.02, 8.08]	
Income,%				<0.001***
≤2,000$	14.01 [9.87, 18.15]	12.93 [9.30, 16.56]	18.47 [12.77, 24.18]	
>2,000$	82.43 [70.65, 94.22]	87.07 [83.44, 90.70]	81.53 [75.82, 87.23]	
BMI				0.003**
Normal weight	26.74 [22.49, 31.00]	26.19 [24.70, 27.68]	28.20 [24.85, 31.55]	
Over weight	35.79 [30.94, 40.64]	37.94 [34.54, 41.33]	30.55 [27.24, 33.87]	
Obesity	37.38 [32.24, 42.51]	35.87 [32.88, 38.85]	41.25 [37.69, 44.81]	
Smoking,%				<0.001***
Current	17.65 [14.40, 20.91]	16.25 [14.10, 18.40]	21.14 [15.44, 26.84]	
Former	28.28 [23.44, 33.12]	25.67 [22.86, 28.47]	34.79 [31.62, 37.95]	
Never	54.06 [46.94, 61.18]	58.08 [55.68, 60.48]	44.07 [37.64, 50.50]	
Alcohol user,%				0.231
Heavy	13.50 [11.71, 15.29]	13.82 [11.71, 15.93]	15.09 [11.38, 18.79]	
Low to moderate	54.43 [45.62, 63.25]	58.81 [52.71, 64.90]	53.29 [49.65, 56.93]	
Never	27.22 [21.94, 32.50]	27.38 [21.53, 33.22]	31.62 [28.41, 34.84]	
Hypertension,%	50.12 [44.52, 55.71]	44.32 [41.67, 46.98]	64.51 [58.96, 70.06]	<0.001***
CKD,%				<0.001**
No CKD	90.11 [78.80, 101.42]	93.67 [92.37, 94.97]	81.26 [78.78, 83.75]	
CKD 3	9.25 [7.84, 10.67]	5.79 [4.48, 7.11]	17.86 [15.22, 20.50]	
CKD 4–5	0.64 [0.38, 0.89]	0.54 [0.25, 0.83]	0.88 [0.32, 1.44]	
eGFR, ml/min/1.73 m^2^	84.36 [83.46, 85.25]	86.86 [85.79, 87.92]	78.17 [76.05, 80.29]	<0.001***
Albumin, g/dl	4.25 [4.24, 4.27]	4.26 [4.23, 4.28]	4.24 [4.21, 4.27]	0.476
Albuminuria, mg/L	33.99 [26.22, 41.76]	31.04 [21.36, 40.73]	41.39 [33.10, 49.68]	0.188
Serum calcium, mg/dl	9.46 [9.43, 9.48]	9.44 [9.41, 9.47]	9.49 [9.43, 9.54]	0.189
Serum phosphorus, mg/dl	3.80 [3.76, 3.83]	3.79 [3.75, 3.83]	3.81 [3.76, 3.85]	0.593
HbA1c,%	5.77 [5.72, 5.83]	5.72 [5.65, 5.78]	5.91 [5.84, 5.99]	<0.001***
FBG, mg/dl	108.32 [106.40, 110.24]	106.62 [104.56, 108.67]	112.34 [107.30, 117.39]	0.072
FBI, pmol/L	74.37 [66.31, 82.42]	75.10 [64.98, 85.22]	72.64 [61.49, 83.79]	0.751
TC, mmol/L	5.06 [5.03, 5.09]	5.08 [5.02, 5.14]	5.01 [4.93, 5.10]	0.362
TG, mmol/L	1.81 [1.74, 1.88]	1.77 [1.69, 1.86]	1.90 [1.81, 1.99]	0.052*
HDL-C, mmol/L	1.41 [1.40, 1.43]	1.43 [1.41, 1.45]	1.37 [1.34, 1.41]	0.004**
LDL-C, mmol/L	2.99 [2.95, 3.04]	3.00 [2.93, 3.07]	2.98 [2.93, 3.03]	0.721

Continuous variables are presented as the mean [95% CI], category variables are presented as the proportion [95% CI].

AAC, abdominal aortic calcification; BMI, body mass index; CKD, chronic kidney disease; eGFR, estimated glomerular filtration rate; HbA1c, glycated hemoglobin; FBG, fasting blood glucose; FBI, fasting blood insulin; TC, total cholesterol; TG, triglycerides; HDL-C, high-density lipoprotein cholesterol; LDL-C, low-density lipoprotein cholesterol.

* *P* value < 0.05, **P value < 0.01, ***P value < 0.001.

### Association between BRI and AAC

**Figure 2:**
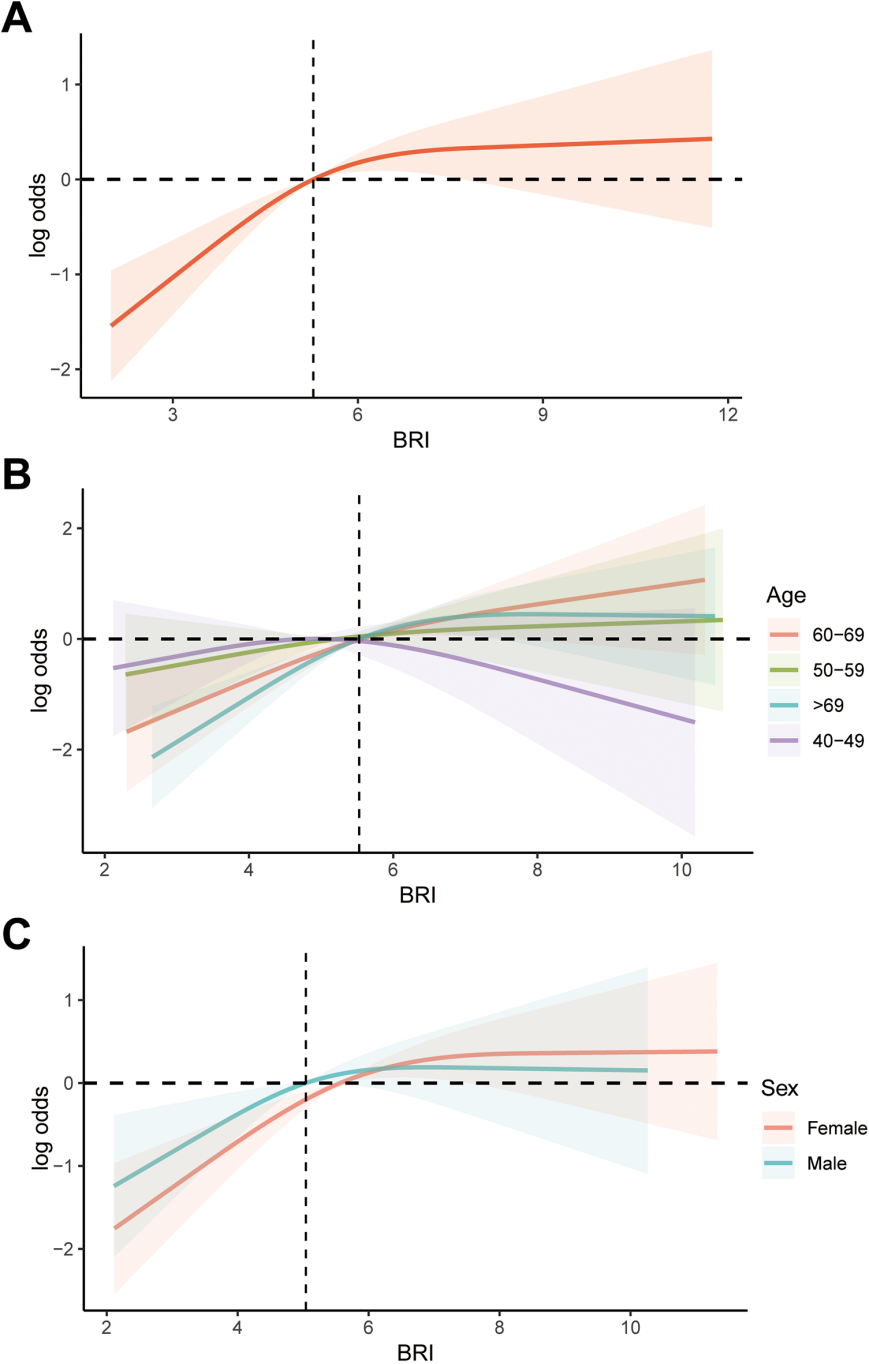
RCS analysis of the association between BRI and AAC. **(A)** Overall analysis; **(B)** subgroup analysis stratified by age; **(C)** subgroup analysis stratified by age gender. RCS, restricted cubic splines; BRI, body roundness index; AAC, abdominal aortic calcification.

In this study, we utilized weighted multivariable logistic regression to investigate the relationship between BRI and AAC. We found that after adjusting for confounding variables, an increase of one unit in BRI was associated with a 22% higher risk of AAC (OR: 1.22; 95% CI: 1.02, 1.46). Additionally, compared to the BRI Q1 group, the risk of AAC in the BRI Q2, Q3, and Q4 groups was 1.86 (95% CI: 1.23, 2.80), 1.83 (95% CI: 1.27, 2.64), and 2.48 (95% CI: 1.47, 4.18) times higher, respectively (Table 2). Additionally, we used RCS to visualize the relationship between BRI and AAC (Figure 2). We found that as BRI increased, the risk of AAC gradually increased. Notably, after BRI exceeded 7.2, the trend of increasing AAC risk slowed down. However, our analysis indicates that there is no significant non-linear association between BRl and AAC, with the non-linear *p* value being 0.12, therefor we didn't carry out segmented logistic regression further. We also analyzed the relationship between BRI and AAC in different age and gender groups. It is worth noting that among individuals aged 40–49, the increase in AAC risk with increasing BRI was not significant. However, in both male and female populations, the risk of AAC increased significantly with higher BRI. However, no non-linear association was found between BRI and AAC among different populations.

**Table 2:** Logistic regression analysis on the association between BRI and AAC.

	Non-adjusted model		Model I		Model II	
	OR [95% CI]	*P* value	OR [95% CI]	*P* value	OR [95% CI]	*P* value
BRI	1.01 [0.98, 1.05]	0.512	0.96 [0.92, 1.02]	0.140	1.22 [1.02, 1.46]	0.032
Q1 (1.43–4.10)	1	–	1	–	1	–
Q2 (4.10–5.27)	1.60 [1.09, 2.35]	0.024*	1.36 [0.93, 1.99]	0.101	1.86 [1.23, 2.80]	0.012*
Q3 (5.27–6.64)	1.44 [1.12, 1.85]	0.013*	1.11 [0.82, 1.48]	0.452	1.83 [1.27, 2.64]	0.003**
Q4 (6.64–13.27)	1.22 [0.92, 1.63]	0.150	0.96 [0.67, 1.38]	0.811	2.48 [1.47, 4.18]	0.002**

Data are presented as OR (95% CI).

Model I adjusted for age, sex, and race/ethnicity.

Model II adjusted for variables in Model I plus income, education levels, BMI, smoking, drinking, hypertension, CKD, eGFR, HbA1c, serum phosphorus, serum calcium, serum albumin.

BRI, body roundness index; AAC, abdominal aortic calcification; BMI, body mass index; CKD, chronic kidney disease; eGFR, estimated glomerular filtration rate; HbA1c, glycated hemoglobin.

* *P* value < 0.05, **P value < 0.01.

## Discussion

We utilized weighted analytical methods to investigate the relationship between the BRI, an indicator of abdominal obesity, and AAC among US residents. We found a positive association suggesting a close relationship between abdominal obesity and AAC.

Due to the limitations of traditional anthropometric measurements, such as BMI or WC, on evaluating of abdominal obesity, BRI has been applied in a large number of previous studies investigating abdominal obesity and its effects on the body. Zhang et al. tracked BRI trends in US adults over 20 years and its association with all-cause mortality. The investigators found that BRI, as a measure reflecting body fat distribution, increased steadily among participants. They also revealed a U-shaped relationship with mortality risk, higher risks were observed at both low and high BRI values compared to mid-range values. Their findings suggest BRI could serve as a valuable noninvasive tool for estimating mortality risk, potentially enhancing public health strategies pending further validation across diverse populations (18). Zhang et al. explored the association between BRI and depression also using NHANES data. BRI was found to correlate positively with depression prevalence among 18,654 adults aged ≥20 years. Adjusted analysis revealed an 8% increase in depression prevalence with each one-unit rise in BRI. The findings suggest BRI could be a valuable tool for predicting depression risk, though causal relationships couldn't be established due to the study's cross-sectional nature and absence of clinical depression diagnoses (19). Another study investigated the correlation between BRI and overactive bladder and confirmed a positive association (OR = 1.15, 95% CI = 1.12–1.17) between BRI and OAB. Moreover, subgroup analyses and ROC curves indicated BRI's superior predictive capability compared to other anthropometric indices, with gender-specific cutoffs (men: 5.151, women: 5.383) suggesting varying susceptibility (20). Experienced measurers in this study used standardized instruments to measure body measurements of participants included in the study. We calculated the Body Roundness Index for all participants using formulas established in previous research.

Considering AAC has a strong predictive capability for cardiovascular adverse events, many studies have conducted detailed research on the risk factors of AAC. A previous study found that participants aged 40 and above showed higher AAC prevalence with age, smoking, diabetes, and hypertension, however, the investigators did not find association between obesity and AAC. The investigators also found that engagement in moderate-to-vigorous physical activity, particularly occupational, was associated with lower AAC scores (*β* = 0.46, *p* < 0.001). Prolonged sedentary behavior correlated with higher AAC scores (*β* = 0.28, *p* = .023). Moreover, subgroup analyses underscored age and hypertension as influencing factors. The findings emphasize the cardiovascular benefits of moderate-to-vigorous physical activity and reduced sedentary time, especially for vulnerable populations (21). Numerous studies have shown that inflammation plays a crucial role in vascular calcification (22, 23), and abdominal obesity can lead to increased systemic inflammation levels in the body. Another study examined the relationship between the systemic immune-inflammation index and AAC, Multivariate logistic regression revealed a positive association, each 100-unit increase in SII correlated with a 4% higher risk of severe AAC. Participants in the highest SII quartile faced a 47% greater risk of severe AAC compared to the lowest quartile, with stronger effects observed in adults over 60 years old. These findings suggest SII could enhance AAC prevention strategies in the US adult population (24). Cai et al. explored the relationship between Life's Essential 8 (LE8), a measure of cardiovascular health, and AAC in adults aged ≥40 years. They found that higher LE8 scores correlated with lower AAC scores and prevalence, especially severe AAC. Adjustment for confounders showed significant associations, with nicotine exposure, blood glucose, and blood pressure components of LE8 demonstrating notable impacts (25). Currently, there is limited research on the relationship between body shape indices specific to abdominal obesity and AAC, and the relationship between waist circumference and AAC has not been reported. A previous cross-sectional study analyzed the association between body shape index (ABSI) and abdominal AAC using data from 3,140 participants aged 40–80 years from the 2013–2014 NHANES. The study found that higher ABSI was significantly associated with an increased risk of AAC (39.8% vs. 23.7%, *P* < 0.001), with multivariate logistic regression and ROC curves confirming that ABSI was independently linked to AAC and had favorable discriminant ability. However, ABSI is not specifically designed for abdominal obesity. In this study, we investigated the relationship between the BRI, a measure specifically for abdominal obesity, and AAC (26). In this study, we conducted a cross-sectional study to investigate the association between BRI and AAC in a large sample of middle-aged and elderly individuals. Abdominal obesity is characterized by an excess of visceral fat, which secretes pro-inflammatory cytokines such as TNF-α, IL-6, and CRP (27). These inflammatory mediators contribute to chronic low-grade inflammation, which promotes endothelial dysfunction and vascular inflammation (28). This inflammatory environment can lead to the activation of vascular smooth muscle cells and the promotion of their differentiation into osteoblast-like cells, which contributes to the calcification of the arterial walls. Visceral fat in abdominal obesity increases the production of ROS. Oxidative stress damages endothelial cells and promotes the accumulation of advanced glycation end-products (AGEs) in the vascular tissue. AGEs and oxidative stress can stimulate the expression of calcification-related proteins such as osteopontin and matrix Gla-protein, which contribute to vascular calcification (29, 30). This study may complement previous research on risk factors associated with AAC.

There are some limitations in the present study should be addressed, firstly, this study is a cross-sectional study, therefore, it is not possible to determine a causal relationship between BRI and AAC. More prospective studies are still needed. Secondly, all participants enrolled in this study were from the United States, so further research is needed to determine whether the findings of this study can be generalized to other countries and ethnic groups. Third, among all NHANES study cycles, only the 2013–2014 cycle included data related to AAC. Compared to other NHANES studies, this study had a limited amount of available research data. Therefore, the conclusions of this study require further confirmation through future works. Lastly, although our study focuses on AAC, it is essential to recognize that AAC differs significantly from other types of arterial calcification. AAC specifically refers to calcification within the abdominal aorta, which may have different pathophysiological mechanisms and clinical implications compared to calcification in other arteries, such as those in the coronary or cerebral circulation. Cardiovascular and cerebrovascular calcification are associated with distinct risk factors and disease processes, which may not be fully captured by assessing AAC alone.

## Conclusion

In the present cross-sectional study enrolling participants from NHANES, we found that BRI, a novel measurement for evaluating abdominal obesity, was positively associated with AAC among US middle-aged and elderly participants. More attention should be paid to abdominal obesity for overall vascular health.

## Data Availability

The original contributions presented in the study are included in the article/Supplementary Material, further inquiries can be directed to the corresponding author.

## References

[1] Bartstra JW Mali W Spiering W de Jong PA. Abdominal aortic calcification: from ancient friend to modern foe. Eur J Prev Cardiol. (2021) 28:1386–91. 10.1177/2047487320919895 34647579

[2] Szulc P. Abdominal aortic calcification: a reappraisal of epidemiological and pathophysiological data. Bone. (2016) 84:25–37. 10.1016/j.bone.2015.12.004 26688274

[3] Golledge J. Abdominal aortic calcification: clinical significance, mechanisms and therapies. Curr Pharm Des. (2014) 20:5834–8. 10.2174/1381612820666140212195309 24533938

[4] Nadanaka S Kitagawa H. EXTL2 Controls liver regeneration and aortic calcification through xylose kinase-dependent regulation of glycosaminoglycan biosynthesis. Matrix Biol. (2014) 35:18–24. 10.1016/j.matbio.2013.10.010 24176719

[5] Raynor WY Borja AJ Zhang V Kothekar E Lau HC Ng SJ Assessing coronary artery and aortic calcification in patients with prostate cancer using (18)F-sodium fluoride PET/computed tomography. PET Clin. (2022) 17:653–9. 10.1016/j.cpet.2022.07.009 36229106

[6] Mazziotti G Tupputi U Ferrante G Guglielmi G. Abdominal aortic calcification as a marker of relationship between atherosclerosis and skeletal fragility. J Clin Densitom. (2020) 23:539–42. 10.1016/j.jocd.2020.05.001 32536435

[7] Desai MY Cremer PC Schoenhagen P. Thoracic aortic calcification: diagnostic, prognostic, and management considerations. JACC Cardiovasc Imaging. (2018) 11:1012–26. 10.1016/j.jcmg.2018.03.023 29976300

[8] Jayalath RW Mangan SH Golledge J. Aortic calcification. Eur J Vasc Endovasc Surg. (2005) 30:476–88. 10.1016/j.ejvs.2005.04.030 15963738

[9] Tao L Miao L Guo YJ Liu YL Xiao LH Yang ZJ. Associations of body roundness index with cardiovascular and all-cause mortality: nHANES 2001–2018. J Hum Hypertens. (2024) 38:120–7. 10.1038/s41371-023-00864-4 37752175

[10] González-Muniesa P Mártinez-González MA Hu FB Després JP Matsuzawa Y Loos RJF Obesity. Nat Rev Dis Primers. (2017) 3:17034. 10.1038/nrdp.2017.34 28617414

[11] Tomiyama AJ. Stress and obesity. Annu Rev Psychol. (2019) 70:703–18. 10.1146/annurev-psych-010418-102936 29927688

[12] Vekic J Zeljkovic A Stefanovic A Jelic-Ivanovic Z Spasojevic-Kalimanovska V. Obesity and dyslipidemia. Metab Clin Exp. (2019) 92:71–81. 10.1016/j.metabol.2018.11.005 30447223

[13] Després JP Lemieux I. Abdominal obesity and metabolic syndrome. Nature. (2006) 444:881–7. 10.1038/nature05488 17167477

[14] Geiker NRW Astrup A Hjorth MF Sjödin A Pijls L Markus CR. Does stress influence sleep patterns, food intake, weight gain, abdominal obesity and weight loss interventions and vice versa? Obes Rev. (2018) 19:81–97. 10.1111/obr.12603 28849612

[15] Carey DG. Abdominal obesity. Curr Opin Lipidol. (1998) 9:35–40. 10.1097/00041433-199802000-00008 9502333

[16] Fang H Berg E Cheng X Shen W. How to best assess abdominal obesity. Curr Opin Clin Nutr Metab Care. (2018) 21:360–5. 10.1097/mco.0000000000000485 29916924 PMC6299450

[17] Thomas DM Bredlau C Bosy-Westphal A Mueller M Shen W Gallagher D Relationships between body roundness with body fat and visceral adipose tissue emerging from a new geometrical model. Obesity (Silver Spring). (2013) 21:2264–71. 10.1002/oby.20408 23519954 PMC3692604

[18] Zhang X Ma N Lin Q Chen K Zheng F Wu J Body roundness index and all-cause mortality among US adults. JAMA Netw Open. (2024) 7:e2415051. 10.1001/jamanetworkopen.2024.15051 38837158 PMC11154161

[19] Zhang L Yin J Sun H Dong W Liu Z Yang J The relationship between body roundness index and depression: a cross-sectional study using data from the national health and nutrition examination survey (NHANES) 2011–2018. J Affect Disord. (2024) 361:17–23. 10.1016/j.jad.2024.05.153 38815765

[20] Zhang Y Song J Li B Wu Y Jia S Shu H Association between body roundness index and overactive bladder: results from the NHANES 2005–2018. Lipids Health Dis. (2024) 23:184. 10.1186/s12944-024-02174-1 38867211 PMC11167800

[21] Sheng C Huang W Wang W Lin G Liao M Yang P. The association of moderate-to-vigorous physical activity and sedentary behaviour with abdominal aortic calcification. J Transl Med. (2023) 21:705. 10.1186/s12967-023-04566-w 37814346 PMC10563258

[22] Durham AL Speer MY Scatena M Giachelli CM Shanahan CM. Role of smooth muscle cells in vascular calcification: implications in atherosclerosis and arterial stiffness. Cardiovasc Res. (2018) 114:590–600. 10.1093/cvr/cvy010 29514202 PMC5852633

[23] Bessueille L Magne D. Inflammation: a culprit for vascular calcification in atherosclerosis and diabetes. Cell Mol Life Sci. (2015) 72:2475–89. 10.1007/s00018-015-1876-4 25746430 PMC11113748

[24] Xie R Liu X Wu H Liu M Zhang Y. Associations between systemic immune-inflammation index and abdominal aortic calcification: results of a nationwide survey. Nutr Metab Cardiovasc Dis. (2023) 33:1437–43. 10.1016/j.numecd.2023.04.015 37156667

[25] Cai Z Liu Z Zhang Y Ma H Li R Guo S Associations between life’s essential 8 and abdominal aortic calcification among middle-aged and elderly populations. J Am Heart Assoc. (2023) 12:e031146. 10.1161/jaha.123.031146 38063150 PMC10863763

[26] Li W Wang Z Li M Xie J Gong J Liu N. Association between a body shape index and abdominal aortic calcification in general population: a cross-sectional study. Front Cardiovasc Med. (2022) 9:1091390. 10.3389/fcvm.2022.1091390 36704474 PMC9871763

[27] Cavalcante RBM Leão L Tavares ABW Lopes KG Kraemer-Aguiar LG. Fat distribution and its correlation with insulin resistance, androgen markers, and proinflammatory cytokines in polycystic ovary syndrome. Horm Metab Res. (2024). 10.1055/a-2386-9281 39226924

[28] Machi JF Altilio I Qi Y Morales AA Silvestre DH Hernandez DR Endothelial c-myc knockout disrupts metabolic homeostasis and triggers the development of obesity. Front Cell Dev Biol. (2024) 12:1407097. 10.3389/fcell.2024.1407097 39100099 PMC11294153

[29] Martins MSA Carneiro WF Monteiro KS Souza SP Vianna A Murgas LDS. Metabolic effects of physical exercise on zebrafish (Danio rerio) fed a high-fat diet. J Comp Physiol B. (2024). 10.1007/s00360-024-01577-x 39085644

[30] Chooi YC Ding C Magkos F. The epidemiology of obesity. Metab Clin Exp. (2019) 92:6–10. 10.1016/j.metabol.2018.09.005 30253139

